# Attentional processing of pain faces and other emotional faces in chronic pain–an eye-tracking study

**DOI:** 10.1371/journal.pone.0252398

**Published:** 2021-05-28

**Authors:** Janosch A. Priebe, Claudia Horn-Hofmann, Daniel Wolf, Stefanie Wolff, Michael Heesen, Katrin Knippenberg-Bigge, Philip Lang, Stefan Lautenbacher

**Affiliations:** 1 Department of Physiological Psychology, Otto-Friedrich-University of Bamberg, Bamberg, Germany; 2 Department of Neurology, MRI, Center for Interdisciplinary Pain Management–Rise-uP, Technical University of Munich, Munich, Germany; 3 Department of Anesthesiology and Pain Therapy, Sozialstiftung Bamberg, Bamberg, Germany; National Institutes of Health, UNITED STATES

## Abstract

Altered attentional processing of pain-associated stimuli–which might take the form of either avoidance or enhanced vigilance–is thought to be implicated in the development and maintenance of chronic pain. In contrast to reaction time tasks like the dot probe, eye tracking allows for tracking the time course of visual attention and thus differentiating early and late attentional processes. Our study aimed at investigating visual attention to emotional faces in patients with chronic musculoskeletal pain (N = 20) and matched pain-free controls (N = 20). Emotional faces (pain, angry, happy) were presented in pairs with a neutral face for 2000 ms each. Three parameters were determined: First fixation probabilities, fixation durations (overall and divided in four 500 ms intervals) and a fixation bias score as the relative fixation duration of emotional faces compared to neutral faces. There were no group differences in any of the parameters. First fixation probabilities were lower for pain faces than for angry faces. Overall, we found longer fixation duration on emotional compared to neutral faces (‘emotionality bias’), which is in accord with previous research. However, significant longer fixation duration compared to the neutral face was detected only for happy and angry but not for pain faces. In addition, fixation durations as well as bias scores yielded evidence for vigilant-avoidant processing of pain faces in both groups. These results suggest that attentional bias towards pain-associated stimuli might not generally differentiate between healthy individuals and chronic pain patients. Exaggerated attentional bias in patients might occur only under specific circumstances, e.g., towards stimulus material specifically relating to the specific pain of the patients under study or under high emotional distress.

## 1. Introduction

Alterations in attentional processing of pain-associated stimuli (e.g., words or pictures relating to pain) are thought to be implicated in the development and maintenance of chronic pain [1]. The occurrence of attentional bias for pain-related information–i.e., preferential attentional processing of pain-related stimuli compared to neutral stimuli–has been confirmed by three meta-analyses [2–4]. Interestingly, this holds for both pain patients and healthy individuals; two of the three meta-analyses [3, 4] found significantly greater bias in patients compared to controls only for one specific stimulus type, namely sensory pain words.

Critically, these meta-analyses only included investigations utilizing reaction time tasks like the dot-probe paradigm as measure of attentional processing. One major disadvantage of these measures is that attentional focus is assessed only at one specific time point at the end of stimulus presentation so that attentional allocation including its shifting during the presentation interval is not captured. However, especially the shifting might be of importance in order to reveal distinct attentional allocation patterns like a `vigilant-avoidant pattern’. This pattern is defined by initial vigilance to a threatening stimulus followed by avoidance of the same stimulus at a later stage and has been shown to be associated with anxiety [5, 6].

In view of these considerations, the eye-tracking paradigm has gained more and more importance in psychological pain research in recent years. Eye-tracking devices trace the time course of visual attention by almost continuously recording eye movements, thus allowing for differentiating between early and late stages of attentional processing. There are to date a few eye-tracking studies, which investigated attention to pain-associated stimuli in patients with chronic pain, and all of them provided evidence for enhanced vigilance to these stimuli in patients compared to controls [7–13]. Some of these studies found differences between patients and controls mainly regarding the initial fixation, with patients showing more frequent or faster initial fixations on pain-related stimuli [10–12], indicating that group differences might be most pronounced in the early phases of attentional processing. In contrast, two investigations, which analyzed the time course in more detail by dividing the 2000 ms presentation interval in four 500 ms segments [8, 9], observed enhanced vigilance in the patient group only in the later stage of processing. In a recent study in a chronic pain sample [14], attentional bias to anger and pain faces emerged during the middle stage (500–1000 ms) and lasted throughout the 3000 ms presentation interval; however, the authors did not include a control group so that the regular eye gaze pattern cannot be inferred.

Taken together, previous findings do not provide evidence for a vigilant-avoidant pattern of attentional processing in pain patients; in contrast, a few studies indicate that patients might actually dwell only longer on pain-related stimuli than controls. In line with these findings, a recent longitudinal study in a chronic pain sample found that difficulties to disengage from injury-related pictures were predictive of higher pain and higher interference six months later [15].

Considering this evidence, it might be assumed that disengagement from threatening stimuli in the later stages of attentional processing (i.e., the avoidant part in the ‘vigilant-avoidant’ pattern) is even adaptive in the context of pain. Prevention of this sane attentional avoidance in chronic pain might then lead to prolonged dwelling on pain cues which might in turn initiate or worsen pain symptoms. In line with this reasoning, a previous study conducted in our lab [16] corroborated a vigilance-avoidance pattern in pain-free individuals when viewing pain faces.

The present study aimed at comparing the time course of attentional processing of happy, angry, and pain faces in pain patients and pain-free controls, using our previously developed paradigm [16]. This paradigm used a free-viewing task as we were specifically interested in gaze behavior without extrinsic motivation due to response requirements, comparable to real life situations when unrelated people are observed when displaying pain. We tried our best to combine the strengths of several previous studies by a) using four distinct emotional categories in order to separate attentional processing of pain stimuli from attentional processing of other emotionally salient stimuli (positively and negatively valenced); b) using photographs of emotional faces as universally relevant emotional stimuli; c) specifically investigating the time course of attentional engagement by dividing the presentation interval in four segments of 500 ms; and d) using a homogeneous, well diagnosed and medically precisely described sample of chronic pain patients and a strictly matched control group.

We assumed that controls would display a tendency towards vigilant-avoidant processing of pain faces as in our previous study. In contrast, we hypothesized that pain patients would show difficulties to disengage from pain faces, with group differences becoming increasingly obvious during later stages of processing.

## 2. Materials and methods

### 2.1 Subjects

#### 2.1.1. Patients

Patients with primary diagnoses of musculoskeletal pain (neck pain, upper back pain, and low back pain) or fibromyalgia lasting for a minimum of 6 months before participation were recruited among outpatients starting to attend a multimodal four-week pain management program at the outpatient unit for pain therapy of the Sozialstiftung Bamberg (Bamberg, Germany). Headaches (migraine, tension-type headache or non-specified headache) were allowed as secondary diagnosis. Exclusion criteria were other predominant pain diagnoses, surgical interventions within the last year and severe mental disorders.

Between January 2012 and February 2013, 94 patients attended the program. Thereof 41 patients met criteria for participation in the study. Twenty of the 41 patients agreed to participate in the study, representing a recruitment rate of 48.8%. Testing took place at a mean of 9.7 days (SD = 5.7) after start of the pain management program. The age of the chronic pain patients ranged between 23 and 57 years (mean age = 42.7, SD = 9.7, female: N = 10). Most of the patients had suffered from chronic pain for 2–5 years (N = 8) or even longer than 5 years (N = 8), whereas the remaining four patients reported a pain duration of 1–2 years (N = 2) or below 1 year (N = 2), respectively.

The distribution of pain diagnoses in our patient sample is shown in Tables 1 and 2 provides an overview of patients`medication. Patients were not asked to pause analgesic medication on the day of testing to assure their compliance and minimize interference with the ongoing pain therapy.

**Table 1:** Pain types in chronic pain patients (n = 20).

DIAGNOSIS	TOTAL	PERCENT
**Chronic back pain**, thereof	**11**	**55%**
Neck pain	2	
Upper back pain	1	
Low back pain	6	
Neck pain and low back pain	1	
**Chronic back pain and headache**, thereof	**4**	**20%**
Neck pain	2	
Neck pain and low back pain	2	
**Chronic back pain, fibromyalgia, and headache**, thereof	**2**	**10%**
Neck pain	1	
Neck pain and low back pain	1	
**Fibromyalgia**	**2**	**10%**
**Fibromyalgia and headache**	**1**	**5%**

Note. Diagnoses made by physician in charge.

**Table 2:** Medication of chronic pain patients.

MEDICATION	TOTAL	PERCENT
**Analgesics**	**17**	**85%**
**on demand**, thereof	5	
Nonsteroidal antiphlogistics	1	
Nonopioid analgesics	1	
Nonsteroidal antiphlogistics and nonopioid analgesics	2	
Nonsteroidal antiphlogistics and opioid analgesics	1	
**on demand in combination with antidepressants**, thereof	1	
Nonsteroidal antiphlogistics	1	
**as prescribed**, thereof	3	
Nonsteroidal antiphlogistics	1	
Nonsteroidal antiphlogistics and opioid analgesics	1	
Nonsteroidal antiphlogistics, nonopioid analgesics, and opioid analgesics	1	
**as prescribed in combination with antidepressants**, thereof	8	
Nonsteroidal antiphlogistics	4	
Nonopioid analgesics	2	
Nonsteroidal antiphlogistics and nonopioid analgesics	1	
Nonopioid analgesics and opioid analgesics	1	
**Antidepressants**	**2**	**10%**
**None**	**1**	**5%**

Note. Analgesics consumed as prescribed were taken at least once a day

#### 2.2.2. Controls

Twenty healthy, pain-free controls (female: N = 11; age: 21–56, M = 43.4, SD = 8.4) were matched to the patients based on age and sex by selecting them from a larger sample of healthy participants. For each patient, we selected an “experimental control twin” with the same sex and a minimal difference in age (0–3 years) from our pool of healthy controls; *t*-tests confirmed that the two groups did not differ regarding age (*t<1*). The pain-free subjects were initially recruited by advertisement at the University of Bamberg and in the local newspaper. None of them was taking any central-nervous medication or had consumed alcohol at least 24 h prior to the test session according to self-report. Exclusion criteria (assessed by a telephone interview) included all acute or chronic diseases.

#### 2.2.3. Ethics

The experimental procedure was approved by the ethics committee of the University of Bamberg. All patients and controls provided written informed consent and received monetary compensation (30 €) for their participation.

### 2.2 Apparatus and stimulus material

Photographs of male and female human faces were extracted from the ‘Montréal Pain and Affective Face Clips’ [17] by creating snapshots at the point of apex expression [18]. We used photographs of pain, angry, happy, and neutral facial expressions. Photographs were transformed from colored into monochromatic to match stimuli regarding hue, brightness, and saturation (which might influence emotional reactions) [18]. The suitability and validity of this set of pictures was confirmed in our previous eye-tracking studies [16, 19] and two other previous studies in our lab [18, 20]. Furthermore, pictures extracted from the same set were also used in three eye-tracking studies referred to in the introduction [10, 11, 16].

For our study, we created 64 pairs of pictures (horizontally aligned). Forty-eight of those pairs (16 for each emotion) consisted of one picture depicting a neutral facial expression and one picture depicting an emotional or painful expression (happy, angry, or pain faces). In these neutral-happy, neutral-anger and neutral-pain pairs, the screen position (left-right) of the neutral picture was randomized to eliminate position effects. As control condition, 16 further pairs consisted of two neutral faces.

All pictures were 7.8 cm wide and 6.1 cm high. The distance between the two pictures forming a pair was 4.8 cm. Stimuli were presented vertically centered against a black background on a 19 in. monitor with a resolution of 1280x1024 pixels. Stimulus presentation and registration of ocular movements were accomplished by an Interactive-Mind system being composed of a desktop-PC with Intel-Processor, a 19 in. LED-screen and the monocular eye-tracking-system EyegazeEdge^TM^ (LC Technologies, Inc., Virginia, USA). In order to measure the eye’s orientation, this system uses the corneal reflection of an infrared light source (corneal reflex method) captured by a camera which is placed below the computer screen. Eye movements were recorded with a sampling rate of 60 Hz and an accuracy of 0.4°. Gaze direction was accepted as fixation if participants’ gaze did not deviate more than 0.7° from the center of the actual fixation for at least 100ms. For stimulus presentation and registration of ocular movements the system was driven by the software NYAN 2^XT^ (version 2.3.3, Interactive Minds GmbH, Dresden, Germany).

### 2.3 Questionnaires

As we aimed at a broad coverage of different cognitive and emotional processing styles relating to pain, we used a set of three questionnaires which are related but neither theoretically nor empirically redundant. This set consisted of the established German versions of the Pain Catastrophizing Scale (PCS) [21, 22], the Pain Anxiety Symptoms Scale (PASS) [23, 24], and the Pain Vigilance and Awareness Questionnaire (PVAQ) [24, 25].

The PCS [21, 22] was developed as a measure of catastrophizing related to pain. It contains 13 items (e.g., ‘I worry all the time about whether the pain will end’). Each item is evaluated on a five-point scale. For further analyses, we used the combined sum score. Total PCS scores range from 0 to 52. According to the user manual, a total PCS score of 30 represents a clinically relevant level of catastrophizing.

The PASS [23, 24] was designed to measure pain-related anxiety across cognitive, behavioral, and physiological domains. It is composed of 4 subscales: cognitive anxiety, escape/avoidance, fearful appraisal, and physiological anxiety. The items are rated on a 6-point scale. For further analyses, the combined sum score of the PASS (40 items) was used which ranges from 0 to 200.

The PVAQ [25, 26] was developed as a comprehensive measure of attention to pain and has been validated for use in acute pain, chronic pain and non-clinical samples [26, 27]. It consists of 16 items (e.g., ‘I am quick to notice changes I pain intensity’) with each item assessed on a six-point scale. For further analyses, the combined sum score of the PVAQ was used, as advised in the literature. PVAQ sum scores range from 0 to 80.

All questionnaires demonstrated good internal consistency (PCS: Cronbach`s α = 0.94; PASS: Cronbach`s α = 0.92; PVAQ: Cronbach`s α = 0.77); values of Cronbach`s α were very similar to those reported for the original English versions [21, 23, 25].

### 2.4 Procedure

After giving their informed consent, participants were seated 70 cm in front of the screen of the computer controlling the eye-tracker. An orthogonal prolongation of the nasion should target the center of the screen. Next, the participants ran through an automatic calibration procedure for relating eye gaze and screen positions. For that purpose, participants were instructed to follow a dot with their eyes, which occurred on different screen positions. After this, the main experiment was started.

At the beginning of each trial, a fixation cross was presented in the center of the screen for 500ms. After the fixation cross had disappeared, one of the 64 picture pairs appeared for 2000ms. Next the screen turned black for 2000ms before the next trial began with the fixation cross. The instruction given to participants was to keep their eyes on the fixation cross at the beginning of each trial and after that to ‘look naturally at the screen’.

All participants performed 64 trials; picture pairs were presented in the same random order for all subjects. After the eye-tracking procedure, the participants were asked to complete the PCS, PASS, and PVAQ (see 2.3). The whole data collection lasted about 20 minutes.

### 2.5 Primary data analysis and parameters

For the purpose of analyzing the gaze behavior, the presented pictures were defined as areas of interest (AOI) which consisted in squares of 7.8 cm x 6.1 cm framing the facial pictures.

In accord with our previous publication [16], three parameters were extracted. The first parameter of interest was the *probability of first fixations*, which either fell into the area of interest (AOI) with the emotional/painful face or in the AOI with the concurrently displayed neutral face. Secondly, we analyzed the time course over the 2000ms of presentation of how the *fixation durations* were distributed between the AOIs associated with the emotional or pain faces on the one hand and the neutral faces over time. For that purpose, the whole presentation time was subdivided into four time epochs: 0-500ms, 500-1000ms, 1000-1500ms and 1500-2000ms. As a third parameter, a *fixation bias score* was computed, which was defined as difference between the fixation time for emotional/painful faces and the fixation time for neutral faces within each stimulus category. We refrained from using specific methods for identifying and excluding outliers, as no conspicuous values could be detected by visual inspection and descriptive analyses.

### 2.6 Statistical analysis

Data referring to the probability of the first fixation were subjected to a split-plot ANOVA with the between-subject factor ‘group’ (patients vs. controls) and the within-subject factor ‘stimulus class’ (happy vs. angry vs. pain. vs. neutral).

Furthermore, the time course of attentional processing of the different emotional/pain faces was analyzed by computing analyses a) based on the absolute fixation durations and b) based on the fixation bias scores. The effects on absolute fixation duration were analyzed by three split-plot ANOVAs with the between-subject factor ‘group’ (patients vs. controls) and the within-subject factors ‘epoch’ (0-500ms vs. 500-1000ms vs. 1000-1500ms vs. 1500-2000ms) and ‘stimulus class’ (neutral vs. emotional) which were run separately for happy, angry, and pain faces. The effects on the fixation bias scores were analyzed by one split-plot ANOVA with the between-subject factor ‘group’ (patients vs. controls) and the within-subject factors ‘epoch’ (0-500ms vs. 500-1000ms vs. 1000-1500ms vs. 1500-2000ms) and ‘stimulus class’ (happy vs. angry vs. pain). By this analysis, we intended to compare directly between the three emotional picture classes the course of fixation durations (relative to the neutral pictures) over time.

Significance level was set to α = 0.05. Adjusting degrees of freedom with Greenhouse-Geisser correction was necessary in case of violation of sphericity. For post-hoc testing of ANOVA-effects, we used Bonferroni-corrected t-tests for dependent samples. For F-tests, partial eta squared (η^2^) (0.01: small effect; 0.06: medium effect; 0.14: large effect) is reported as an estimate of effect size; Cohen’s s d (0.20: small effect; 0.50: medium effect; 0.80: large effect) is reported to describe effect size for paired comparisons [28].

## 3. Results

### 3.1 Sample characteristics

Compared to the control subjects, patients reported significantly higher pain catastrophizing, pain anxiety and pain vigilance (PCS: patients: M = 31.0, SD = 17.32 vs. controls: M = 14.4, SD = 8.65; PASS: patients: M = 105.7, SD = 35.36 vs. controls: M = 59.4, SD = 30.34; PVAQ: patients: M = 47.9, SD = 8.59 vs. controls: M = 36.5, SD = 9.84), all p’s < 0.001. The sociodemographic data, pain diagnoses, pain durations and medications are already given in 2.1 (for details see Tables 1 and 2).

### 3.2 Gaze behavior

The results regarding the gaze behavior are reported in two sections: (i) findings regarding initial attentional allocation (i.e., probability of first fixation) and (ii) findings regarding attentional allocation over the 2000 ms of presentation (i.e., time course of absolute fixation durations and fixation bias scores).

#### 3.2.1. Probability of first fixation

**Fig 1:**
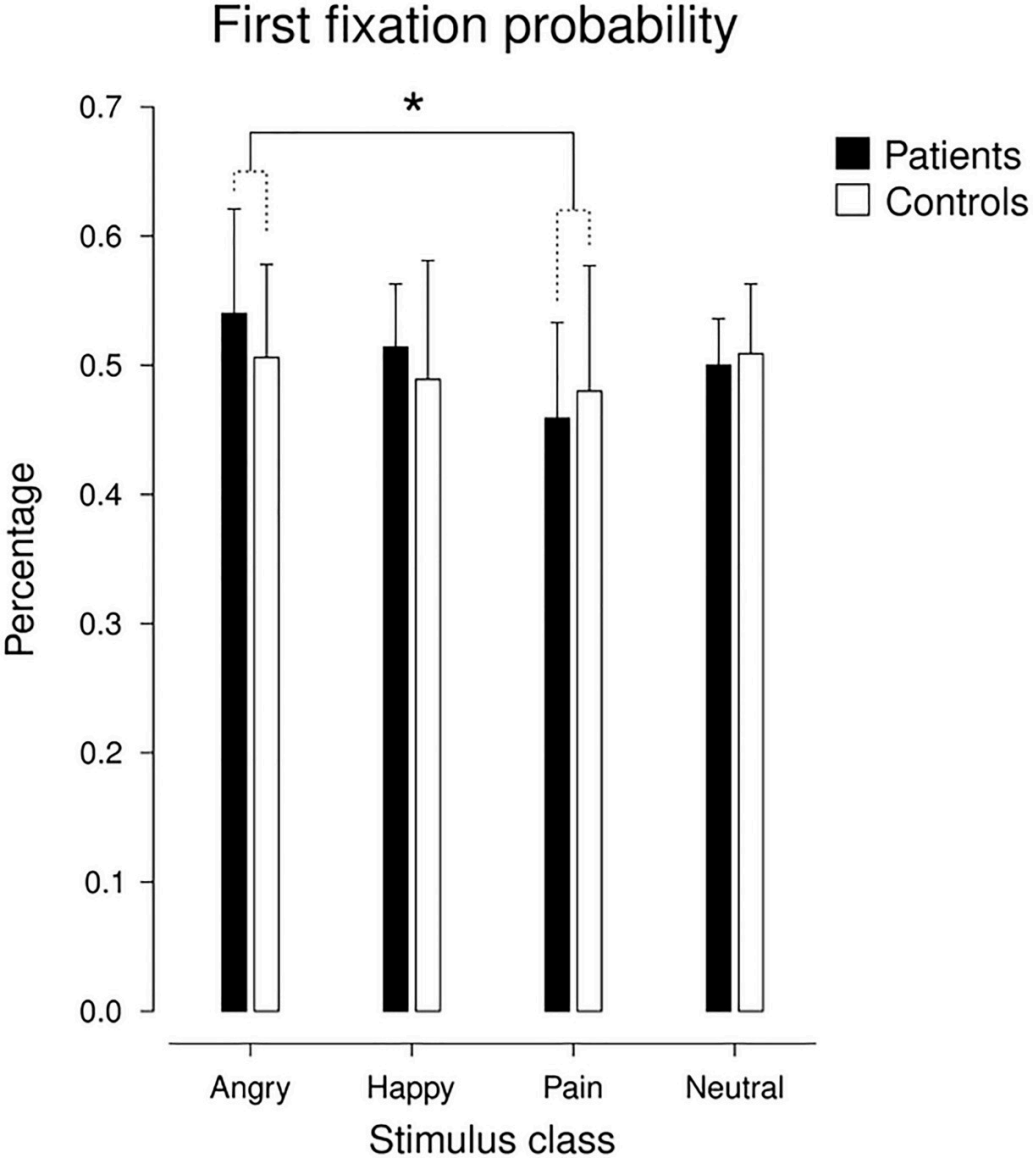
Means and SDs of the first fixation probabilities for angry, happy, and pain faces vs. paired neutral faces both for pain patients and controls. * *p* < 0.05.

For the *probability of first fixation*, we detected no significant main effect of ‘group’ (F(1,38) = 0.752, p = 0.391, ɳ^2^ = 0.019) and no ‘group’ x ‘stimulus class’ interaction, indicating no differences between patients and controls. However, the ANOVA yielded a significant main effect of ‘stimulus class’ (F(3,114) = 3.085, p = 0.030, ɳ^2^ = 0.075). Post-hoc tests yielded a significant difference between pain and anger, with first fixation probabilities being lower for painful than for angry faces (t(39) = 3.160, p = 0.003, d = 0.65); all other paired comparisons failed to pass significance (Bonferroni-corrected α = 0.008). Descriptive statistics of first fixation probabilities for the two groups and the four stimulus classes are depicted in Fig 1.

#### 3.2.2. Time course of attentional allocation

**Fig 2:**
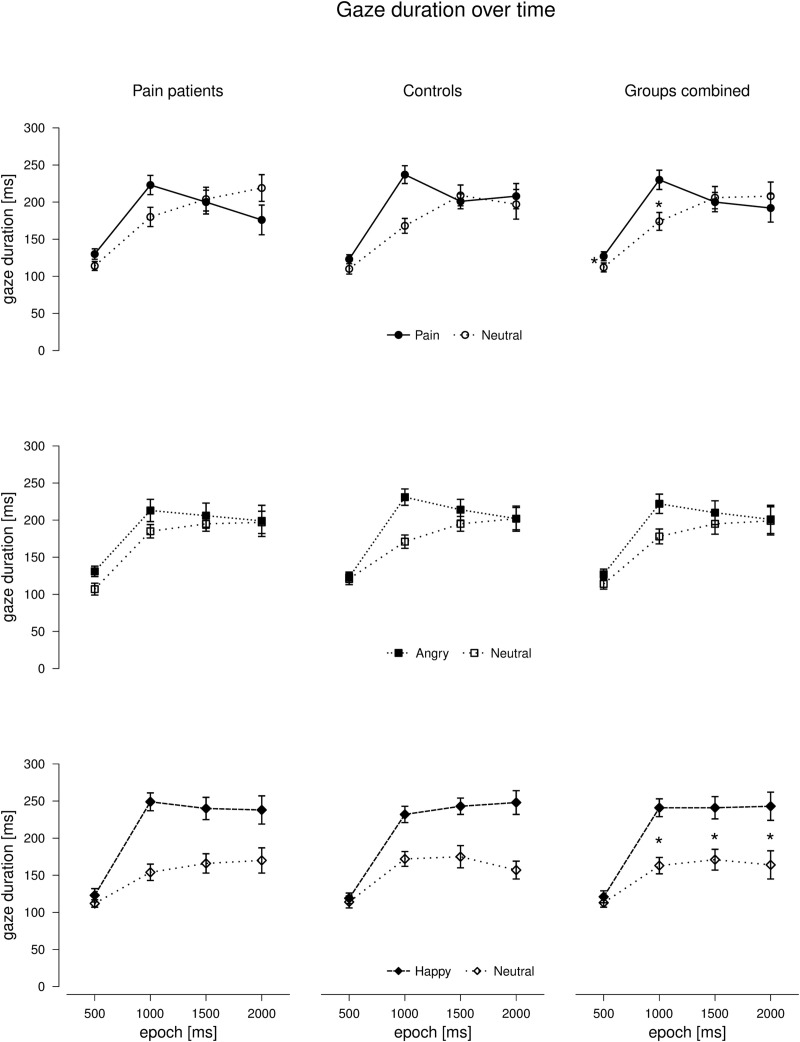
Means (± SE) of the absolute fixation durations for the three emotional stimulus classes (pain, angry and happy faces) compared to neutral faces over 4 epochs of 500 ms during stimulus presentation separately for pain patients and controls and for both groups combined. Asterisks indicate significant differences.

*3*.*2*.*2*.*1*. *Fixation duration*. Results of the ANOVAS on the absolute fixation durations (separately for angry, happy, and pain faces) are summarized in Table 3. Importantly, none of the three ANOVAs yielded a significant effect of ‘group’ (no main effect and no interactions), indicating that the pattern of attentional allocation was similar for patients and controls. As depicted in Fig 2, longer fixation durations were found in epochs 2 to 4 compared to epoch 1 for all three emotional stimulus classes (main effect of ‘epoch’ in all ANOVAs). Additionally, there was a main effect of ‘stimulus class’ for happy and angry faces, indicating preference of the emotional vs. the neutral face across epochs; in contrast, this effect was not detected for pain faces. Instead, an interaction between ‘stimulus class’ and ‘epoch’ emerged for pain faces: As can be seen in Fig 2, pain faces were preferred over neutral faces in epoch 1 (t(39) = 2.431, p = 0.020, d = 0.49) and epoch 2 (t(39) = 4.697, p < 0.001, d = 1.00), whereas there were no significant differences between pain faces and neutral faces in the later epochs (epoch 3: t(39) = 0.404, p = 0.688, d = 0.08; epoch 4: t(39) = 0.692, p = 0.493, d = 0.11). For happy faces, there was also a ‘stimulus class’ x ‘epoch’ interaction, with preference for happy over neutral faces emerging after epoch 1 (epoch 1: t(39) = 1.453, p = 0.154, d = 0.28; epoch 2–4: all p’s < 0.001) (Bonferroni-corrected α = 0.013). It is to mention that the main effect of `stimulus’ for angry faces was significant but rather small when compared to the effect for happy faces as indicated by effect sizes (see Table 3).

**Table 3:** Test statistics of the ANOVAs for the effects of group (patients vs. controls), stimulus class (emotional vs. neutral), and epoch (0–500 ms vs. 500–1000 ms vs. 1000–500 ms vs. 1500–2000 ms) on fixation durations for each of the three emotional categories.

	Effect	*F*	*p*	*η*^*2*^
Angry	Group	0.085	0.772	0.002
	**Stimulus**	**4.622**	**0.038**	**0.108**
	**Epoch**	**261.727**	**<0.001**	**0.873**
	Group x Stimulus	0.056	0.813	0.001
	Group x Epoch	0.017	0.997	< 0.001
	Stimulus x Epoch	1.802	.151	0.045
	Group x Stimulus x Epoch	0.704	0.551	0.018
Happy	Group	0.011	0.919	<0.001
	**Stimulus**	**36.330**	**<0.001**	**0.489**
	**Epoch**	**304.496**	**<0.001**	**0.889**
	Group x Stimulus	0.090	0.765	0.002
	Group x Epoch	0.488	0.691	0.013
	**Stimulus x Epoch**	**6.151**	**0.001**	**0.139**
	Group x Stimulus x Epoch	0.728	0.537	0.019
Pain	Group	0.008	0.928	<0.001
	Stimulus	1.745	0.194	.044
	**Epoch**	**214.302**	**<0.001**	**0.849**
	Group x Stimulus	0.998	0.324	0.026
	Group x Epoch	0.637	0.592	0.016
	**Stimulus x Epoch**	**4.986**	**0.008**	**0.116**
	Group x Stimulus x Epoch	0.931	0.428	0.024

Bold script indicates significant effects (*p* < 0.05).

**Fig 3:**
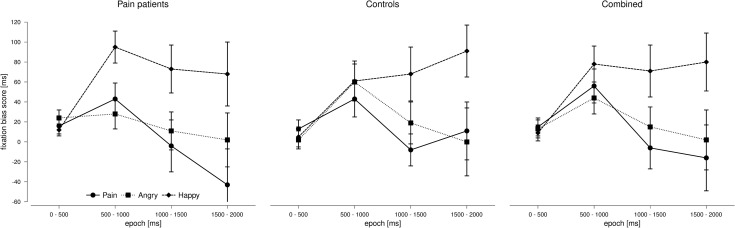
Means (± SE) of the fixation bias scores for the three emotional stimulus classes (pain, angry, and happy faces) over 4 epochs of 500ms during stimulus presentation separately for pain patients and controls and for both groups combined.

*3*.*2*.*2*.*2*. *Fixation bias scores*. Results of the ANOVA on the *fixation bias scores* are summarized in Table 4. Again, there were no significant effects of ‘group’ (no main effect and no interactions). As depicted in Fig 3, we found a higher fixation bias score for happy faces relative to angry faces (t(39) = 2.840, p *=* 0.007, d = 0.55) and pain faces (t(39) = 2.886, p = 0.006, d = 0.46) across epochs (main effect of ‘stimulus class’). Independent of stimulus class, fixation bias scores were highest in epoch 2 (see Fig 3); significant differences were observed between epoch 1 and 2 (t(39) = 4.827, p < 0.001, d = 0.93) and between epoch 2 and 3 (t(39) = 3.094, p = 0.004, d = 0.61) but not between epoch 2 and 4 (t(39) = 2.313, p = 0.026, d = 0.47) (Bonferroni-corrected α = 0.008). All other paired comparisons failed to reach significance (all p’s > 0.100).

**Table 4:** Test statistics of the ANOVAs for the effects of group (patients vs. controls), stimulus class (happy vs. angry vs. pain), and epoch (0–500 ms vs. 500–1000 ms vs. 1000–500 ms vs. 1500–2000 ms) on fixation bias scores.

	Effect	*F*	*p*	*η*^*2*^
Fixation bias score	Group	0.323	0.573	0.008
	**Stimulus**	**7.121**	**0.005**	**0.158**
	**Epoch**	**5.382**	**0.005**	**0.124**
	Group x Stimulus	0.401	0.671	0.010
	Group x Epoch	0.727	0.538	0.019
	**Stimulus x Epoch**	**3.633**	**0.014**	**0.087**
	Group x Stimulus x Epoch	0.830	0.548	0.021

Bold script indicates significant effects (*p* < 0.05).

In addition, we detected a significant interaction between ‘stimulus class’ and ‘epoch’: As can be seen in Fig 3, all three emotional categories showed a similar time course in the beginning, with bias scores increasing from epoch 1 to epoch 2. However, different patterns emerged thereafter: We observed a decline in attentional preference for pain faces and angry faces from epoch 2 to epoch 3 (see Fig 3), whereas attentional preference for happy faces remained at a constant high level. Post-hoc t-tests confirmed that there was no change in attentional bias for happy faces from epoch 2 to epoch 3 (t (39) = 0.404, p = 0.688, d = 0.07), whereas attentional bias for pain faces dropped significantly within this time window (t(39) = 3.397, p = 0.002, d = 0.72). For angry faces, the decline in attentional bias failed to reach significance after Bonferroni correction (t(39) = 2.042, p = 0.048, d = 0.35) (Bonferroni-corrected α = 0.017).

### 3.3 Correlation between gaze behavior and questionnaire scores

In order to check for group-specific associations between pain-related psychological variables (pain catastrophizing (PCS), pain anxiety (PASS), pain vigilance (PVAQ)) and gaze behavior, we computed an exploratory correlation analysis between these three questionnaire scores and the 12 fixation bias scores relating to the four time epochs and the three emotional categories (pain, angry, happy) separately for patients and controls. For each group, only one correlation out of 36 passed the level of significance. In the patient group, there was a significant correlation between the fixation bias score for angry faces in epoch 3 and the PASS score (r = 0.525, p = 0.018). In the control group, there was a significant correlation between the fixation bias score for happy faces in epoch 1 and the PVAQ score (r = 0.0450; p = 0.047). These two correlations appear to be random in face of no replications in the others epochs and the high number of further non-significant correlations, and would not persist after Bonferroni correction (corrected α = 0.0014). Thus, we observed no relevant association between psychological questionnaire data and behaviorally assessed attentional bias in the present study.

### 3.4 Summary of results

Taken together, our analyses revealed the following results: (i) Pain patients did not differ from healthy control subjects in any of the parameters describing gaze behavior despite significant differences in all questionnaire scores, which can be supposed to indicate cognitive and emotional pain processing. (ii) Overall, all participants displayed an emotionality bias, i.e., higher fixation duration for emotional compared to neutral faces. (iii) The time course of attentional allocation varied between emotions: Happy faces were preferred over neutral faces across epochs; in contrast, there was a drop in attentional preference for angry and pain faces after epoch 2, which was more pronounced for pain faces.

## 4. Discussion

The objective of the present study was to compare the time course of visual attention to pain faces and other emotional faces between pain-free controls and pain patients with chronic functional, mainly musculoskeletal pain. Our main findings were twofold. First, we found no differences between the two groups in any of the parameters assessing gaze behavior despite enhanced self-reported pain-related anxiety, vigilance and catastrophizing in patients. Second, the three emotional categories differed regarding the time course of attentional allocation: Whereas happy faces were constantly preferred over neutral faces from epoch 2 to epoch 4 (500–2000 ms), there was a drop in preference for angry and painful faces after epoch 2; this pattern was more pronounced for pain than for anger. These main findings will be discussed in detail in the following paragraphs.

Independent of group (patients vs. controls), we found that (1) emotional faces attracted more attention than neutral faces, and (2) happy faces were attentionally preferred over angry and pain faces in the later stages of processing. These findings are in line with the results of our previous study [16] and with several other eye tracking studies either reporting attentional preference for emotional stimuli in general (emotionality bias) [8, 9, 12, 14] or attentional preference for happy faces (positivity bias) [10, 29] in both pain patients and controls.

Our finding that visual attention to pain faces was not altered in chronic pain patients is in contrast to several previous studies using the eye-tracking methodology [7–13]. However, it is in line with recent findings by Mazidi and colleagues [30], who also assessed the time course of attentional processing of pain faces in pain patients and controls and observed no group differences. Thus, although positive results still prevail, there is recently emerging heterogeneity concerning evidence for altered attentional processing in pain patients captured by eye tracking. This is in line with meta-analytical findings that bias to pain-related stimuli in chronic pain patients obtained in studies using a primary task paradigm (dot-probe, Stroop, spatial cueing) is of small effect size and not significantly different from controls with the exception of bias for sensory pain words which seems to be more pronounced in patients [3]. A more recent meta-analysis including only dot-probe investigations [4] was able to confirm significantly stronger bias for sensory pain words in patient samples, with no differences observed for pictorial stimuli or other word types. Thus, group differences in attentional bias to pain-related stimuli are not observed consistently and seem to depend on task parameters like the stimulus type, which somewhat questions a prominent role of biased attention in the etiology of chronic pain. In addition, attentional bias to pain cues in pain patients might not be omnipresent, but only emerge in situations when pain is actually expected or experienced. In line with this reasoning, Jackson and colleagues [31] were able to show that gaze bias to pain-related images increased in a condition where pictures signaled upcoming noxious stimulation. However, the authors observed very similar results in a sample of pain-free controls [32], thus questioning the assumption that group differences might become more visible under such circumstances. Future studies should aim at further clarifying how patient characteristics, stimulus material, and situational threat might contribute to biased attention in chronic pain patients. Conducting larger studies encompassing pain patient samples diverse as regards etiology, chronicity and medication as well as different types of stimuli and experimental settings might be advisable.

The present study also failed to show that patients suffering from chronic pain specifically exhibit mere vigilance to pain cues and little attentional avoidance compared to pain-free individuals as hypothesized in the introduction due to a synopsis of earlier findings. Thus, our own previous study [16] as well as the investigation by Mazidi and colleagues [30] provided evidence for vigilant-avoidant processing of pain faces in healthy, pain-free individuals, suggesting that a vigilant-avoidant pattern is sane and adaptive when viewing pain-related stimuli. In that regard, several other eye-tracking studies reported enhanced vigilance to pain-related stimuli without any late attentional avoidance in pain patients [8, 9, 14]. In accord, a recent longitudinal study in a chronic pain sample showed that difficulties to disengage from injury-related pictures were predictive of worse clinical outcomes six months later [15]. It is still reasonable to assume that the strength of attentional focus on pain depends on the context and therefore may largely be different in chronic pain patients compared to pain-free individuals [33], although our present findings did not corroborate this assumption.

Thus, the question remains why patients did not display difficulties to disengage from pain cues in the present study and also in the study by Mazidi and colleagues [30], whereas they showed this maladaptive attentional pattern in several other studies [8, 9, 14]. First, the stimulus material might play a role; in the current study, in our previous study [16] and also in the study by Mazidi and colleagues [30], photographs of pain faces and emotional faces were used as stimulus material. As humans are evolutionarily prepared to decode facial expressions, pain patients might have fewer difficulties in processing pain faces rapidly and disengaging from them thereafter compared to more multifarious or symbolic stimuli. In line with this reasoning, the two studies supporting sustained vigilance to pain cues in patients used pain-related words [8] or photographs of injuries [9] which might both require more in-depth semantic processing. However, Lee and colleagues [14] observed continuous attentional preference for pain faces throughout the 3000 ms presentation interval in a patient sample, thus contradicting this assumption. Another factor which might influence whether differences between patients and controls are observed is the specific relevance of the pain-associated stimuli for the patient sample. For example, sensory pain words like ‘aching’ or ‘burning’ [8] might be highly relevant to patients as they directly relate to their pain experience. The same holds true for photographs of painful movements [12, 13], which are specifically relevant to patients with musculoskeletal pain. Due to the large heterogeneity of previous studies relating to the pain diagnoses (headache vs. musculoskeletal pain vs. mixed), the stimulus presentation paradigm (primary task vs. passive viewing) and the stimulus material (words vs. pain faces vs. photographs of injuries or situations), the contribution of each of these parameters needs to be clarified in future research.

Finally, it should be mentioned that attentional bias to pain-associated stimuli can be influenced by personal and situational characteristics both in patients and healthy individuals. For example, higher pain catastrophizing was associated with early avoidance of pain faces in healthy subjects [34] and with longer gaze duration on pain faces in pain patients [14]. Similarly, fear of pain/(re)injury influenced the attentional pattern displayed by pain patients in a dot-probe paradigm: Whereas controls and patients with low fear shifted attention away from pain faces, patients with high fear attended towards these stimuli instead [35]. These results might also explain differences between studies: Patient samples with relatively low fear/catastrophizing might show the same attentional pattern as controls while samples with high fear/catastrophizing might behave differently. In this context, it should be considered that patients included in the current study had already been in treatment for 10 days on average; although this might have been too early to detect specific effects of pain therapy, non-specific effects like social support and positive expectations might have led to reduced psychological distress and thus normalized attentional processing.

Some limitations of our study should be considered. First, as already mentioned patients were at the beginning of a multi-dimensional pain therapy and were not asked to pause medication so that we cannot exclude that our results were influenced by treatment effects. Second, our experimental design did not contain response requirements. This may lead to two problems: (i) We cannot make sure that the presented information (pictures of faces) was indeed processed in depth by participants; (ii) attention was investigated during passive viewing but not in the context of goal-directed behavior which might lead to a restriction of ecological validity [36]. Third, our stimulus material showing facial expressions of acute emotions and pain might not have been of specific relevance for mainly back pain patients; thus, we cannot exclude that group differences might have occurred for other types of stimuli (e.g., back pain related words or pictures of back-straining movements).

Taken together, our study revealed no differences between chronic pain patients and healthy individuals regarding visual attention to emotional faces and pain faces despite higher self-reported pain-related catastrophizing, anxiety and vigilance in the patient group. Both groups showed a steady attentional preference for happy faces and a tendency towards vigilant-avoidant processing of angry faces and particularly pain faces. Our findings failed to demonstrate that chronic pain is accompanied by biased attention to pain associated stimuli. Future research should try to identify personal and situational factors potentially contributing to differences between chronic pain patients and healthy individuals.

## Supporting information

S1 Table Descriptive statistics of eye-tracking parameters for both groups. (DOCX)
