# Supplementary material for: Attentional processing of pain faces and other emotional faces in chronic pain–an eye-tracking study
Source: PLoS One. 2021 May 28;16(5):e0252398. doi: 10.1371/journal.pone.0252398 (PMC8162699; doi:10.1371/journal.pone.0252398)
Supplement: S1 Table — (DOCX) [file pone.0252398.s001.docx]

**S1 Table.**

**Descriptive statistics of fixation probabilities for angry, happy, and pain faces in comparison to neutral faces**

|  | **Group** | **M (SD)** | **N** |
| --- | --- | --- | --- |
| **Angry faces** | Patients | 0.54 (0.08) | 20 |
|  | Controls | 0.51 (0.07) | 20 |
| **Happy faces** | Patients | 0.51 (0.05) | 20 |
|  | Controls | 0.49 (0.09) | 20 |
| **Pain faces** | Patients | 0.46 (0.07) | 20 |
|  | Controls | 0.48 (0.10) | 20 |

**Descriptive statistics of fixation bias scores for the three emotional stimulus classes (angry, happy, and pain faces) across the four epochs**

|  | **Group** | **Epoch** | **Fixation bias score (M (SD))** |
| --- | --- | --- | --- |
| **Angry faces** | Patients | 1 (0-500 ms) | 23.95 (37.62) |
|  |  | 2 (500-1000 ms) | 27.60 (66.08) |
|  |  | 3 (1000-1500 ms) | 11.45 (88.61) |
|  |  | 4 (1500-2000 ms) | 1.90 (121.61) |
|  | Controls | 1 (0-500 ms) | 1.65 (41.88) |
|  |  | 2 (500-1000 ms) | 60.15 (78.90) |
|  |  | 3 (1000-1500 ms) | 19.35 (92.40) |
|  |  | 4 (1500-2000 ms) | 0.40 (149.40) |
| **Happy faces** | Patients | 1 (0-500 ms) | 11.80 (28.90) |
|  |  | 2 (500-1000 ms) | 94.90 (71.30) |
|  |  | 3 (1000-1500 ms) | 73.25 (108.47) |
|  |  | 4 (1500-2000 ms) | 68.45 (142.80) |
|  | Controls | 1 (0-500 ms) | 5.40 (45.33) |
|  |  | 2 (500-1000 ms) | 60.50 (87.51) |
|  |  | 3 (1000-1500 ms) | 67.75 (121.91) |
|  |  | 4 (1500-2000 ms) | 91.00 (114.70) |
| **Pain faces** | Patients | 1 (0-500 ms) | 16.30 (38.26) |
|  |  | 2 (500-1000 ms) | 42.70 (69.68) |
|  |  | 3 (1000-1500 ms) | -4.15 (115.74) |
|  |  | 4 (1500-2000 ms) | -42.80 (162.80) |
|  | Controls | 1 (0-500 ms) | 13.15 (38.89) |
|  |  | 2 (500-1000 ms) | 69.05 (79.94) |
|  |  | 3 (1000-1500 ms) | -8.05 (72.92) |
|  |  | 4 (1500-2000 ms) | 10.75 (127.80) |

**Descriptive statistics of fixation duration for the three emotional stimulus classes (angry, happy, and pain faces) and the paired neutral faces across the four epochs**

|  | **Group** | **Epoch** | **Fixation duration in ms (M (SD))** |
| --- | --- | --- | --- |
| **Angry faces** | Patients | 1 (0-500 ms) | 130.80 (28.42) |
|  |  | 2 (500-1000 ms) | 212.70 (59.05) |
|  |  | 3 (1000-1500 ms) | 206.30 (70.63) |
|  |  | 4 (1500-2000 ms) | 199.10 (79.77) |
|  | Controls | 1 (0-500 ms) | 123.00 (29.91) |
|  |  | 2 (500-1000 ms) | 231.35 (45.45) |
|  |  | 3 (1000-1500 ms) | 213.90 (61.22) |
|  |  | 4 (1500-2000 ms) | 202.20 (90.60) |
| **Neutral faces**  **(angry-neutral trials)** | Patients | 1 (0-500 ms) | 106.75 (34.17) |
|  |  | 2 (500-1000 ms) | 184.95 (55.62) |
|  |  | 3 (1000-1500 ms) | 194.90 (69.31) |
|  |  | 4 (1500-2000 ms) | 197.10 (83.06) |
|  | Controls | 1 (0-500 ms) | 121.30 (30.14) |
|  |  | 2 (500-1000 ms) | 171.20 (49.79) |
|  |  | 3 (1000-1500 ms) | 194.70 (50.62) |
|  |  | 4 (1500-2000 ms) | 201.85 (73.38) |
| **Happy faces** | Patients | 1 (0-500 ms) | 123.45 (33.42) |
|  |  | 2 (500-1000 ms) | 248.70 (67.53) |
|  |  | 3 (1000-1500 ms) | 239.55 (76.73) |
|  |  | 4 (1500-2000 ms) | 238.40 (92.43) |
|  | Controls | 1 (0-500 ms) | 119.30 (31.36) |
|  |  | 2 (500-1000 ms) | 232.35 (50.71) |
|  |  | 3 (1000-1500 ms) | 242.80 (64.21) |
|  |  | 4 (1500-2000 ms) | 248.05 (77.10) |
| **Neutral faces**  **(happy-neutral trials)** | Patients | 1 (0-500 ms) | 111.60 (24.06) |
|  |  | 2 (500-1000 ms) | 153.80 (50.16) |
|  |  | 3 (1000-1500 ms) | 166.30 (60.33) |
|  |  | 4 (1500-2000 ms) | 170.05 (78.34) |
|  | Controls | 1 (0-500 ms) | 113.90 (34.42) |
|  |  | 2 (500-1000 ms) | 171.85 (45.82) |
|  |  | 3 (1000-1500 ms) | 175.05 (67.11) |
|  |  | 4 (1500-2000 ms) | 156.95 (55.45) |
| **Pain faces** | Patients | 1 (0-500 ms) | 129.95 (30.83) |
|  |  | 2 (500-1000 ms) | 222.80 (60.35) |
|  |  | 3 (1000-1500 ms) | 200.00 (72.16) |
|  |  | 4 (1500-2000 ms) | 175.80 (89.61) |
|  | Controls | 1 (0-500 ms) | 123.20 (27.03) |
|  |  | 2 (500-1000 ms) | 237. 00 (51.64) |
|  |  | 3 (1000-1500 ms) | 200.55 (45.72) |
|  |  | 4 (1500-2000 ms) | 208.20 (76.48) |
| **Neutral faces**  **(pain-neutral trials)** | Patients | 1 (0-500 ms) | 113.70 (26.55) |
|  |  | 2 (500-1000 ms) | 180.15 (51.35) |
|  |  | 3 (1000-1500 ms) | 204.15 (81.65) |
|  |  | 4 (1500-2000 ms) | 218.75 (104.51) |
|  | Controls | 1 (0-500 ms) | 110.10 (35.61) |
|  |  | 2 (500-1000 ms) | 167.90 (39.66) |
|  |  | 3 (1000-1500 ms) | 208.60 (43.12) |
|  |  | 4 (1500-2000 ms) | 197.40 (69.27) |
